# Alterations of the gut microbiota in patients with immunoglobulin light chain amyloidosis

**DOI:** 10.3389/fimmu.2022.973760

**Published:** 2022-10-19

**Authors:** Jipeng Yan, Jin Zhao, Xiaoxuan Ning, Yunlong Qin, Yan Xing, Yuwei Wang, Qing Jia, Boyong Huang, Rui Ma, Changhui Lei, Meilan Zhou, Zixian Yu, Yumeng Zhang, Wei-Feng Guo, Shiren Sun

**Affiliations:** ^1^ Department of Nephrology, Xijing Hospital, Fourth Military Medical University, Xi’an, China; ^2^ Department of Geriatrics, Xijing Hospital, Fourth Military Medical University, Xi’an, China; ^3^ Xijing Hypertrophic Cardiomyopathy Center, Department of Ultrasound, Xijing Hospital, Xi’an, China; ^4^ School of Electrical Engineering, Zhengzhou University, Zhengzhou, China; ^5^ State Key Laboratory of Oncology in South China, Collaborative Innovation Center for Cancer Medicine, Sun Yat-sen University Cancer Center, Guangzhou, China

**Keywords:** AL amyloidosis, amyloid, gut dysbiosis, gut microbiota, immunoglobulin light chain amyloidosis

## Abstract

**Background:**

Emerging evidence revealed that gut microbial dysbiosis is implicated in the development of plasma cell dyscrasias and amyloid deposition diseases, but no data are available on the relationship between gut microbiota and immunoglobulin light chain (AL) amyloidosis.

**Methods:**

To characterize the gut microbiota in patients with AL amyloidosis, we collected fecal samples from patients with AL amyloidosis (n=27) and age-, gender-, and BMI-matched healthy controls (n=27), and conducted 16S rRNA MiSeq sequencing and amplicon sequence variants (ASV)-based analysis.

**Results:**

There were significant differences in gut microbial communities between the two groups. At the phylum level, the abundance of *Actinobacteriota* and *Verrucomicrobiota* was significantly higher, while *Bacteroidota* reduced remarkably in patients with AL amyloidosis. At the genus level, 17 genera, including *Bifidobacterium*, *Akkermansia, and Streptococcus* were enriched, while only 4 genera including *Faecalibacterium*, *Tyzzerella*, *Pseudomonas*, and *Anaerostignum* decreased evidently in patients with AL amyloidosis. Notably, 5 optimal ASV-based microbial markers were identified as the diagnostic model of AL amyloidosis and the AUC value of the train set and the test set was 0.8549 (95% CI 0.7310-0.9789) and 0.8025 (95% CI 0.5771-1), respectively. With a median follow-up of 19.0 months, further subgroup analysis also demonstrated some key gut microbial markers were related to disease severity, treatment response, and even prognosis of patients with AL amyloidosis.

**Conclusions:**

For the first time, we demonstrated the alterations of gut microbiota in AL amyloidosis and successfully established and validated the microbial-based diagnostic model, which boosted more studies about microbe-based strategies for diagnosis and treatment in patients with AL amyloidosis in the future.

## 1 Introduction

Immunoglobulin light chain (AL) amyloidosis is the most common type of systemic amyloidosis, triggered by an underlying plasma cell dyscrasia and involves increased production and release of free immunoglobulin light chains, which form insoluble amyloid fibrils that are deposited in organs (1, 2). The incidence of AL amyloidosis is only 12 cases per million persons per year (3) and advanced multiorgan involvement often leads to delayed diagnosis and poor prognosis (4). Over the last decade, the advent of another plasma cell dyscrasia, multiple myeloma (MM)–derived novel agents such as proteasome inhibitors and daratumumab, has improved the longevity of most AL amyloidosis patients (5, 6). However, several issues remain unaddressed, such as a suboptimal hematological complete response rate, high early mortality, various outcomes depending on the extent and severity of organ damage, and frequently observed treatment-related toxic effects (7). In response to these existing problems, researches are mounting to identify factors that are responsible for the onset and progression of AL amyloidosis and determine the response to treatment in individual patients.

Recently, regarding the relationship between plasma cell dyscrasias and gut microbiota, several studies highlighted an intimate and intricate interaction between gut microbiota and MM (8–10). For example, *Prevotella heparinolytica* was found to promote the progression of MM in Vk*MYC mice by stimulating the differentiation of Th17 cells that inhabited the gut and migrated to the bone marrow (10). Jian and his colleagues (8) demonstrated that nitrogen-cycle bacteria such as *Klebsiella* and *Streptococcus* were considerably abundant in MM, which was probably due to the excessive accumulation of blood urea, and the altered gut microbiota, in turn, contributed to the malignant progression of MM. In addition, gut dysbiosis was observed in recipients of autologous hematopoietic cell transplantation (auto-HCT), which is the first-line therapy in MM or AL amyloidosis patients, and reduced peri-engraftment diversity in fecal samples is linked to poorer overall and progression-free survival in auto-HCT patients (11). Furthermore, the butyrate-produced bacteria *Eubacterium hallii* was more abundant in MM patients with minimal residual disease (MRD)^-^ compared to MRD^+^ patients, which indicated an association between microbial community and treatment responses in MM patients (12).

The important role of gut microbiota in other types of amyloidosis was also preliminarily explored. Among patients with Familial Mediterranean fever (FMF), AA amyloidosis (AAA) was found to have two increased operational taxonomic units (OTUs) in the gut microbiota, and increased indoleamine 2,3-dioxygenase (IDO) activity and higher adiponectin levels (13). Additionally, the serum amyloid-associated (SAA) protein could function as an opsonin, and intestinal epithelial cells could synthesize it in response to the gut microbiota (14, 15). Furthermore, AAA can be induced by a high-fat diet in a mouse model overexpressing hepatic SAA (16), and numerous bacteria can release amyloid-enhancing factors that may be transmitted by ingestion and cross species barriers (17–20). In the autopsy series, approximately all patients with amyloidosis had digestive system involvement (21), and evidence of gut microbiota involvement in the etiology of another amyloid deposition disease, Alzheimer’s disease, is accumulating both in animal models and humans (22–25).

Based on these findings, gut microbiota seems to be a potential candidate for both synthesis of amyloid-enhancing factors and the overproduction of amyloid-related protein in amyloidosis diseases. However, to our best knowledge, no data are available on the relationship between gut microbiota and AL amyloidosis. In this study, we intended to fill this gap in knowledge by characterizing the gut microbial community in patients with AL amyloidosis, identifying specific microbial markers and validating their diagnostic efficacy, and further conducting a preliminary exploration of the relationship between baseline gut microbiota and disease severity, treatment response, and prognosis of AL amyloidosis.

## 2 Materials and methods

### 2.1 Research subjects

The research was conducted in accordance with the PRoBE design concept (prospective specimen collection and retrospective blinded evaluation) (26). This study was approved by the Ethics Committee of Xijing Hospital of the Fourth military medical university (KY20192070), and the participants provided their written informed consent to participate in this study.

Patients who were newly diagnosed with AL amyloidosis between July 2018 and February 2021 in Xijing hospital (Xi’an, China) were screened. The inclusion criteria included patients: I) were biopsy-proven primary systemic AL amyloidosis; II) ≥18 years of age. The exclusion criteria were patients: I) had digestive diseases or systemic diseases such as diabetes and hypertension; II) have been treated with chemotherapy; III) used antibiotics or probiotics within 3 months before sampling; IV) in the period of pregnancy.

Healthy volunteers who were age-, gender-, and body mass index (BMI)-matched with AL amyloidosis patients from the physical examination center of Xijing hospital were enrolled. The inclusion criteria were individuals: I) had a normal value of kidney and liver function, routine blood, feces, and urine tests, fasting blood glucose, blood lipids, and blood pressure; and II) ≥18 years of age. The exclusion criteria were individuals: I) administered antibiotics or probiotics three months before enrollment; II) had a history of any chronic diseases or acute infection.

### 2.2 Clinical data collection

The AL amyloidosis patient registration form was designed to record the demographic and clinical characteristics at kidney biopsy including gender, age, height, weight, blood pressure, medical history, pathological results, biochemical indexes, and therapies. All indicators above were uniformly tested and issued by the Laboratory Department of Xijing Hospital and extracted from the patient medical record system. Clinical organ involvement was assessed according to international consensus criteria (27), and the Mayo 2012 staging system was used for stratification (28).

### 2.3 Follow-up and survival outcomes

The final follow-up date was March 31, 2022. Overall survival (OS) was defined as the time from diagnosis to death or the last follow-up. Patients who were alive at the last follow-up were censored at that date. Progression-free survival (PFS) was defined as the period from treatment initiation to disease progression, relapse, or death from any cause.

### 2.4 Therapeutic strategy and response evaluation

All patients received subcutaneous bortezomib at a dose of 1.3 mg per square meter of body-surface area, and dexamethasone at a dose of 40 mg orally or intravenously once weekly for 28 days each cycle. The assessment of hematological and organ response was performed according to the validated response criteria published by the International Society of Amyloidosis (29, 30). A ≥25% eGFR decrease was considered as the criterion for renal progression. All response assessment was performed on an intent-to-treat (ITT) basis.

### 2.5 Fecal samples collection and bacterial taxon identification

Each individual provided a fresh tail stool sample at 06:30–08:30 hours. Fecal samples were quickly placed in sterile specimen tubes, and transferred to a -80°C cryogenic refrigerator for further analysis. DNA extraction was carried out as previously described by our team (31).

### 2.6 Polymerase chain reaction (PCR), miseq sequencing, and sequence data processing

DNA libraries were constructed, and the sequencing was performed on an Illumina MiSeq platform by Shanghai Mobio Biomedical Technology Co. Ltd., China. The 16S ribosomal RNA (rRNA) gene sequence that targeted the V3-V4 region was applied to identify the bacterial taxon, which was detailed described in a previously published article by our team (31). Original Illumina read data for all samples were stored in the NCBI Sequence Read Archive (SRA) database under accession number PRJNA825339 and PRJNA574226.

### 2.7 Bioinformatics and statistical analysis

Amplicon sequence variants (ASVs) were identified with the DADA2 algorithm. The representative sequences for each ASV were annotated using the SILVA reference database (SSU138). QIIME feature classifier was used for species annotation, which was the species annotation plug-in of the QIIME 2 analysis process, and adopted the classify-sklearn algorithm. Alpha diversity metrics (ACE estimator, Chao 1 estimator, Shannon-Wiener diversity index and Simpson diversity index) were assessed by using Mothur v1.42.1. The non-parametric Mann-Whitney U test was used to test for significant differences between two groups. A comparison of multiple groups was done using a nonparametric Kruskal-Wallis test. Bray-Curtis, weighted UniFrac, unweighted UniFrac, and Jaccard-binary dissimilarity were calculated in QIIME. Principal coordinate analysis (PCoA) plots and PERMANOVA which were used to test for statistical significance between the groups using 10,000 permutations were generated in R (version 3.6.0) package vegan 2.5-7. The linear discriminant analysis (LDA) effect size (LEfSe) (32) was used to detect taxa with differential abundance among groups (lefse 1.1, https://github.com/SegataLab/lefse). A heatmap plot of the key ASVs identified by random forest models was generated by using the ‘pheatmap’ package of the R program. Probability of disease (POD) index was defined as the ratio between the number of randomly generated decision trees that predicted sample as “AL amyloidosis” and that of healthy controls. The identified optimal set of ASVs was finally used for the calculation of POD index for both the training and the testing cohort. A receiver operating characteristics (ROC) analysis was performed to measure the quality of the classification models by the R software package pROC. PICRUSt2 v2.4.1(https://github.com/picrust/picrust2/wiki) (33) was used to predict functional abundances in the Kyoto Encyclopedia of Genes and Genomes (KEGG) PATHWAY database based on 16S rRNA gene sequences. Spearman’s rank correlation was performed to explore correlations between ASVs and the clinical characteristics of patients with AL amyloidosis.

## 3 Result

### 3.1 Baseline clinical characteristics of patients with AL amyloidosis and healthy controls

**Figure 1:**
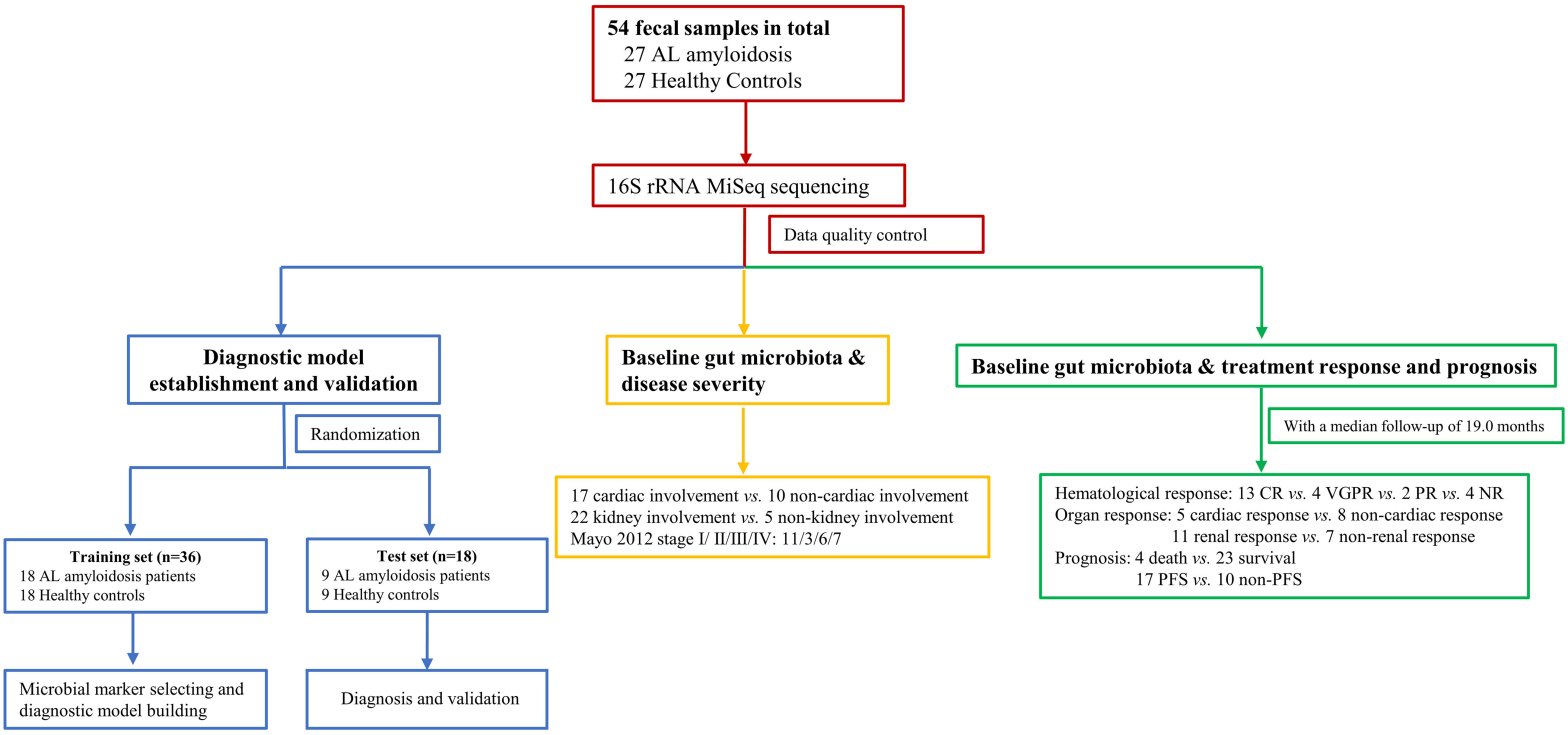
Study design and flow diagram. A total of 54 fecal samples from 27 AL amyloidosis patients and 27 healthy controls were collected. Firstly, we characterized the gut microbiota in patients with AL amyloidosis compared with age-, gender-, and BMI-matched HCs. Secondly, a diagnostic model was established and validated to identify the optimal microbial markers for AL amyloidosis. Furthermore, after a median follow-up of 19.0 months, subgroup analysis also demonstrated some key gut microbial markers were related to organ involvement, mayo 2012 stages, hematological response, organ response, and even prognosis of patients with AL amyloidosis. AL amyloidosis, immunoglobulin Light Chain Amyloidosis; BMI, body mass index; HCs, healthy controls; CR, complete response; VGPR, very good partial response; PR, patial response; NR, no response; PFS, progression-free survival.

We recruited 27 patients with AL amyloidosis and 27 healthy controls (HCs). Process and flow chart of this study were demonstrated in **Figure 1**. The baseline clinical characteristics of these two groups were shown in **Table 1**. Age, gender, and BMI were matched between these two groups. There was also no significant difference between two groups at serum creatinine level, while albumin was considerably lower in patients with AL amyloidosis than that in healthy controls. The median baseline difference between the involved and uninvolved free light chain (dFLC) levels was 142 mg/L (range, 15 to 1201), and the median proteinuria was 2780 mg/24 h (range, 102 to 7905). A total of 18 patients (66.7%) had two or more organs involved; 81.48% of patients had kidney involvement, and 62.96% had heart involvement. The majority of patients (96.29%) were classified as lambda isotype and approximately half of patients (48.15%) had a Mayo 2012 stage of III or higher.

**Table 1:** Demographic and clinical characteristics of the participants at baseline.

Characteristic	AL amyloidosis (n=27)	Healthy control (n=27)	*P* value
Age, mean ± SD, yr	56.78 ± 8.95	53.04 ± 7.17	0.096
Gender (Male/Female)	19/8	14/13	0.264
BMI, mean ± SD, kg/m^2^	22.83 ± 3.77	24.39 ± 1.68	0.057
Albumin, mean ± SD, g/L	29.19 ± 8.12	46.78 ± 3.20	<0.001
Serum Creatinine, mean ± SD, umol/L	73.30 ± 21.49	76.78 ± 10.17	0.451
24-h urinary protein, median (range), mg	2780 (102-7905)	—	
ALP, median (range), IU/L	68 (35-373)	—	
Lambda isotype, no. (%)	26 (96.29%)	—	
dFLC, median (range), mg/L	142 (15-1201)	—	
Involved organs, no. (%)		—	
Kidney Heart Liver Other	22 (81.48%) 17 (62.96%) 2 (7.41%) 21 (77.78%)		
NT-proBNP, median (range), ng/L	890 (30-15398)	—	
cTnT, median (range), ng/L	0.029 (0.004-0.168)	—	
IVST, mean ± SD, cm	13.67 ± 4.67	—	
LVEF, mean ± SD, %	56.74 ± 5.22	—	
Mayo 2012 stage, no. (%)		—	
I II III IV	11 (40.74%) 3 (11.11%) 6 (22.22%) 7 (25.93%)		

BMI, body mass index; ALP, alkaline phosphatase; dFLC, difference in the involved to uninvolved free light chain; NT-proBNP, N-terminal pro-B type natriuretic peptide; cTnT, Troponin T; IVST, inter-ventricular septum thickness; EF, LVEF, left ventricular ejection fraction.

### 3.2 Data quality and changes in gut microbiota diversity of AL amyloidosis

**Figure 2:**
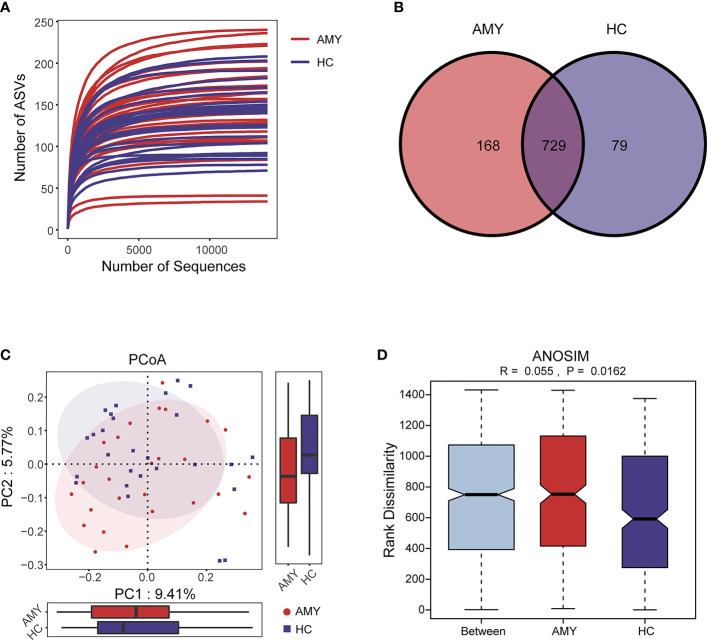
Data quality and gut microbiota diversity in patients with AL amyloidosis and HCs. **(A)** The rarefaction curve revealed the number of ASVs of each sample at different sequencing quantities. **(B)** The Venn diagram displaying overlap between the two groups showed that 729 of the 976 ASVs were shared between the AMY and HCs. A total of 168 of the 976 ASVs were unique to AMY. **(C)** β diversity based on PCoA analysis (Jaccard-binary distances) showed the fecal microbial communities in patients with AMY group and the HCs were clustered separately, indicating a good differentiation in taxonomic composition **(** **Supplementary Table 3** **)**. **(D)** ANOSIM based on Jaccard-binary distances also showed that there were significant differences between the two groups (R = 0.055, *p* = 0.0162, **Supplementary Table 3**). AMY, patients with AL amyloidosis; HCs, healthy controls; ASVs, amplicon sequence variants; PCoA, principal coordinate analysis.

The rarefaction curve revealed the number of ASVs of each sample at different sequencing quantities, which indicated that the amount of sequencing data is large enough to reflect the vast majority of microbial information in the samples when the curve approaches plateau **(** **Figure 2A** **)**. In total, 3,629,337 usable raw reads were obtained from 54 stool samples. After quality filtering and assembly of overlapping paired-end reads, 1,892,625 high-quality reads were generated and 976 ASVs were obtained. The average number of sequences per sample was 67,209 ± 20,151 (range 35,925–120,795, **Supplementary Table 1**). A total of 729 ASVs were shared among the two groups according to the Venn diagram, while 168 ASVs were unique for AL amyloidosis, and 79 ASVs were specific for HCs **(** **Figure 2B** **).** No significant differences in community richness (estimated by Chao and ACE indices) and diversity (measured by Shannon and Simpson indices) were observed between AL amyloidosis and healthy controls **(** **Supplementary Table 2** **)**. The gut microbial communities in patients with AL amyloidosis and the HCs were clustered separately, indicating a marked differentiation in taxonomic composition based on Jaccard-binary distances (Adonis, *p* = 0.0155, **Figure 2C**; **Supplementary Table 3**). In the ANOSIM analysis based on Jaccard-binary distances (**Figure 2D**; **Supplementary Table 3**), the difference between the two groups was significantly greater than that within the groups, further confirming the value of comparison between these two groups (R = 0.055, *p* = 0.0162).

### 3.3 Changes in gut microbiota at the phylum and the genus levels in AL amyloidosis

Based on the taxonomic classification of the ASVs, the relative microbial abundance of 54 samples at different levels (phylum, class, order, family, and genus) was identified. The phylum and genus levels of fecal microbial composition in each sample from two groups were shown in **Supplementary Figures 1A, B**.

**Figure 3:**
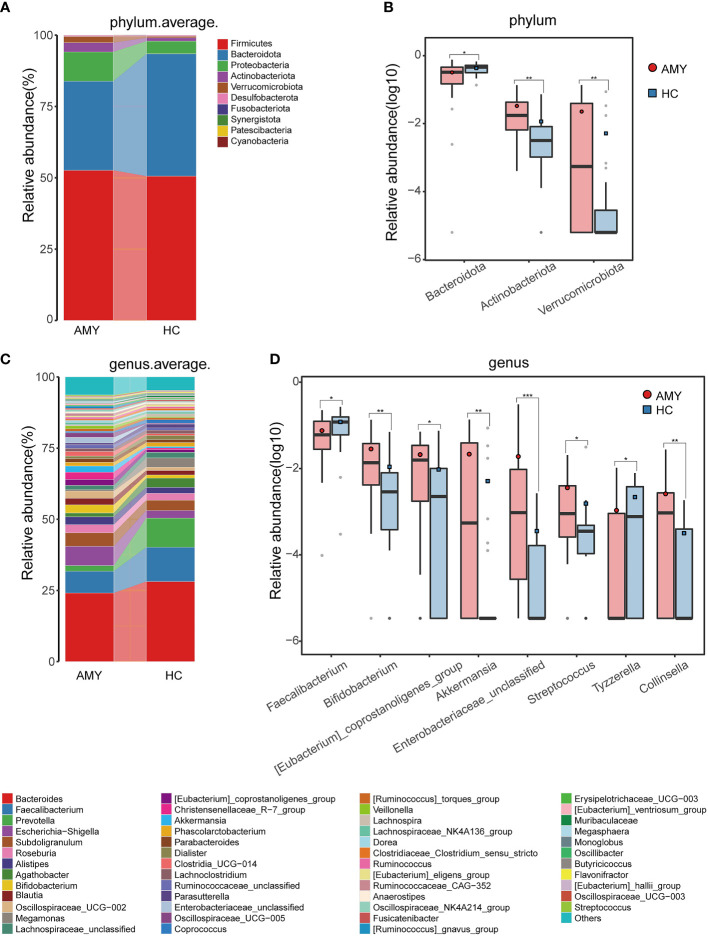
Composition and comparison of gut microbiomes in AMY (n = 27) and HCs (n = 27). Composition of the gut microbiota at the **(A)** phylum and **(C)** genus levels in AMY versus HCs. The significant different microbial community at the phylum level **(B)** and genus level **(D)** in AMY and HCs. The average abundance of each bacterium is depicted as the mean ± SE. P-values were calculated by a Wilcoxon rank-sum test and are shown in the **Supplementary Tables 4, 5**. *P < 0.05, **P < 0.01, ***P < 0.001. AMY, patients with AL amyloidosis; HCs, healthy controls.

At the phylum level, *Firmicutes*, *Bacteroidota*, and *Proteobacteria* were the three dominant populations in two groups, accounting for up to 90% of sequences on average **(** **Figure 3A** **)**. Compared with HCs, *Actinobacteriota* and *Verrucomicrobiota* were enriched (3.22% *vs.* 1.15%, 2.20% *vs.* 0.52%, respectively) in patients with AL amyloidosis, while *Bacteroidota* was considerably reduced in patients with AL amyloidosis (31.20% *vs.* 42.85%, all *p* < 0.05, **Figure 3B**; **Supplementary Table 4**).

At the genus level, *Bacteroides*, *Faecalibacterium*, and *Escherichia-Shigella* in AL amyloidosis, while *Bacteroides*, *Faecalibacterium*, and *Prevotella* in the HCs, were three dominant populations in two groups, each accounting for up to 5% of the sequences on average **(** **Figure 3C** **).** By comparison, we observed that a total of 21 genera had significantly different abundance between the two groups, and the relative abundance of the top 8 genera was presented in **Figure 3D**. Specifically, six genera, namely, *Bifidobacterium*, *[Eubacterium]_coprostanoligenes_group*, *Akkermansia*, *Enterobacteriaceae_unclassified, Streptococcus*, and *Collinsella* were enriched in the AL amyloidosis group (all *p* < 0.05), whereas *Faecalibacterium* and *Tyzzerella* were remarkably decreased in AL amyloidosis group (all *p* < 0.05, **Figure 3D**; **Supplementary Table 5**).

### 3.4 Phylogenetic profiles of the gut microbial communities in AL amyloidosis

**Figure 4:**
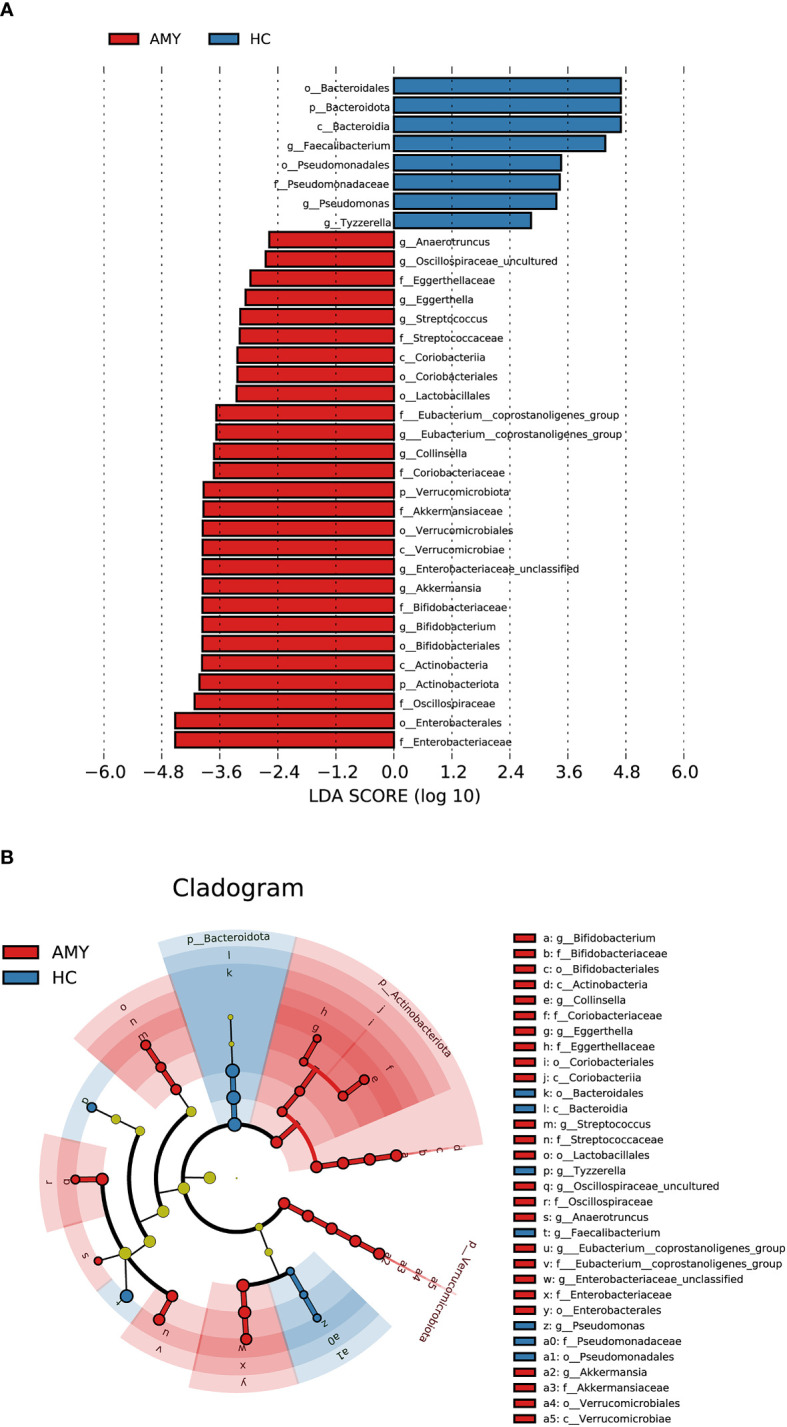
LEfSe analysis based on ASV characterizations of gut microbiota in AMY (n = 27) and HCs (n = 27). **(A)** Histogram of LDA scores calculated for selected taxa showed significant differences in microbe type and abundance between AMY (red) and HCs (blue). LDA scores on a log10 scale are indicated at the bottom. The significance of the microbial marker increases with the LDA score. **(B)** Cladogram generated by the LEfSe method showed the phylogenetic distribution of gut microbiomes associated with AMY and HCs. Nodes in red indicate taxa that were enriched in AMY compared to those in HCs, while nodes in blue indicate taxa that were enriched in HCs compared to those in AMY. Only the taxa having a *p* < 0.05 (Wilcoxon rank-sum test) and LDA >2.5 are shown in the figure legend (**Supplementary Table 6**). AMY, patients with AL amyloidosis; HCs, healthy controls; ASVs, amplicon sequence variants; LEfSe, linear discriminant analysis effect size; LDA, linear discriminant analysis; *p*, phylum; *c*, class; *o*, order; *f*, family; *g*, genus.

In addition to the differences at the above different levels, we analyzed gut microbiota using the LEfSe approach to identify the specific taxa associated with AL amyloidosis. The histogram of LDA value distribution **(** **Figure 4A** **)** revealed that 35 microbial biomarkers clearly distinguished patients with AL amyloidosis and HCs (LDA > 2.5, all *p* < 0.05, **Supplementary Table 6**). Meanwhile, a cladogram **(** **Figure 4B** **)** depicting the fecal microbial structure and prevalent bacteria revealed the significant alterations in taxa between AL amyloidosis patients and HCs. Compared with the healthy group, patients with AL amyloidosis had a markedly higher abundance of several bacterial taxon chains within the phylum *Verrucomicrobiota* and *Actinobacteriota*. Specifically, *p-Verrucomicrobiota.c-Verrucomicrobiae.o-Verrucomicrobiales.f-Akkermansiaceae.g-Akkermansia*, *p-Actinobacteriota.c-Actinobacteria.o-Bifidobacteriales.f-Bifidobacteriaceae.g-Bifidobacterium*, *p-Actinobacteriota.c-Coriobacteriia.o-Coriobacteriales.f-Coriobacteriaceae.g-Collinsella*, as well as genus *Eggerthella* of family *Eggerthellaceae* in phylum *Bacteroidota*, were enriched in AL amyloidosis. Whereas in phyla *Bacteroidota* and *Proteobacteria*, two taxon clades, *p-Bacteroidota.c-Bacteroidia.o-Bacteroidales* and *o-Pseudomonadales.f-Pseudomonadaceae.g-Pseudomonas*, were substantially less abundant in AL amyloidosis patients (LDA > 2.5, all *p* < 0.05).

### 3.5 Microbial functional alteration in AL amyloidosis

**Figure 5:**
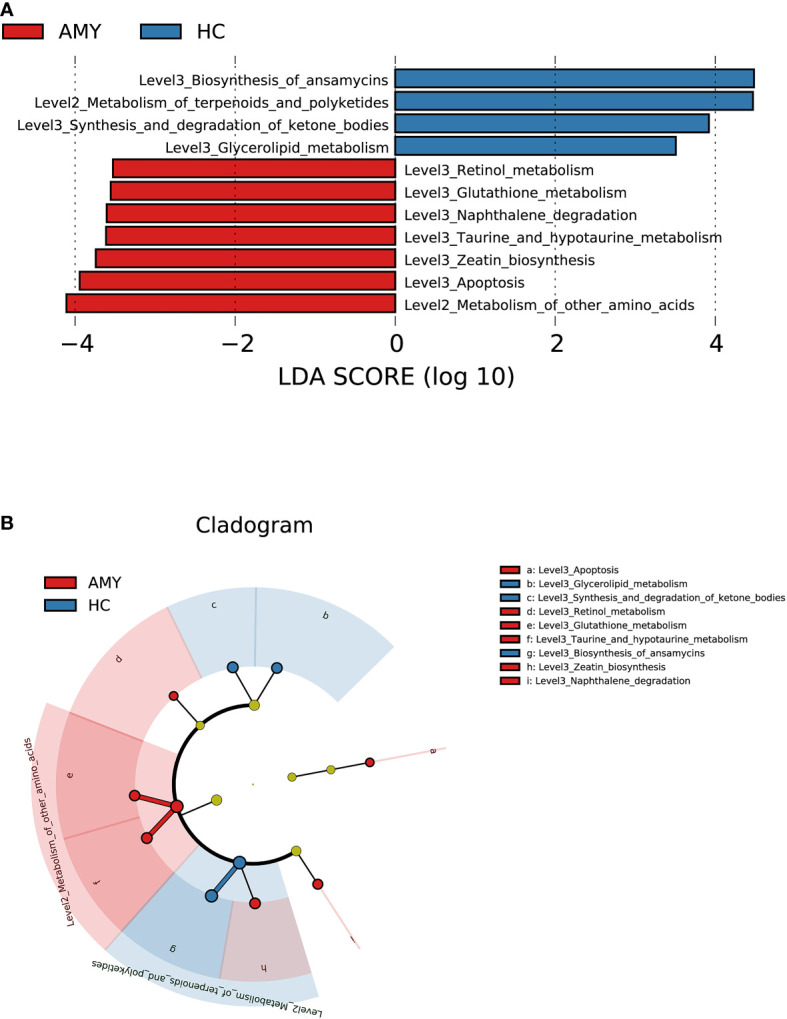
Prediction for differential functional and metabolic alterations of gut microbiota in AL amyloidosis by PICRUSt2 analysis. **(A)** Histogram of LDA scores calculated for selected KEGG pathways showed significant differences in gene functions between AMY (red) and HCs (blue). **(B)** Cladogram generated by the LEfSe method showed the phylogenetic distribution of differential gene functions in AMY and HCs. The default criteria LDA > 2.5 and *p* < 0.05 indicate different KEGG pathways and a higher abundance in one group than in the other (**Supplementary Table 7**). AMY, patients with AL amyloidosis; HCs, healthy controls; LEfSe, linear discriminant analysis effect size; LDA, linear discriminant analysis; KEGG, Kyoto Encyclopedia of Genes and Genomes.

To elucidate the functional and metabolic alterations of gut microbiota between AL amyloidosis and HC groups, PICRUSt2 analysis was used to predict functional abundances based on 16S rRNA gene sequences. We observed a substantial increase in functional abundance of 7 KEGG pathways in patients with AL amyloidosis compared with HCs, including one pathway at level 2, metabolism of other amino acids, and 6 pathways at level 3, namely, retinol metabolism, glutathione metabolism, naphthalene degradation, taurine and hypotaurine metabolism, zeatin biosynthesis, and apoptosis. Whereas, the genes of four pathways including metabolism of terpenoids and polyketides at level 2, and biosynthesis of ansamycins, synthesis and degradation of ketone bodies, glycerolipid metabolism at level 3 were significantly decreased in AL amyloidosis patients (all *p* < 0.05, LDA > 2.5, **Figures 5A, B**, and **Supplementary Table 7**).

### 3.6 Correlations between significantly different ASVs and clinical characteristics in AL amyloidosis

**Figure 6:**
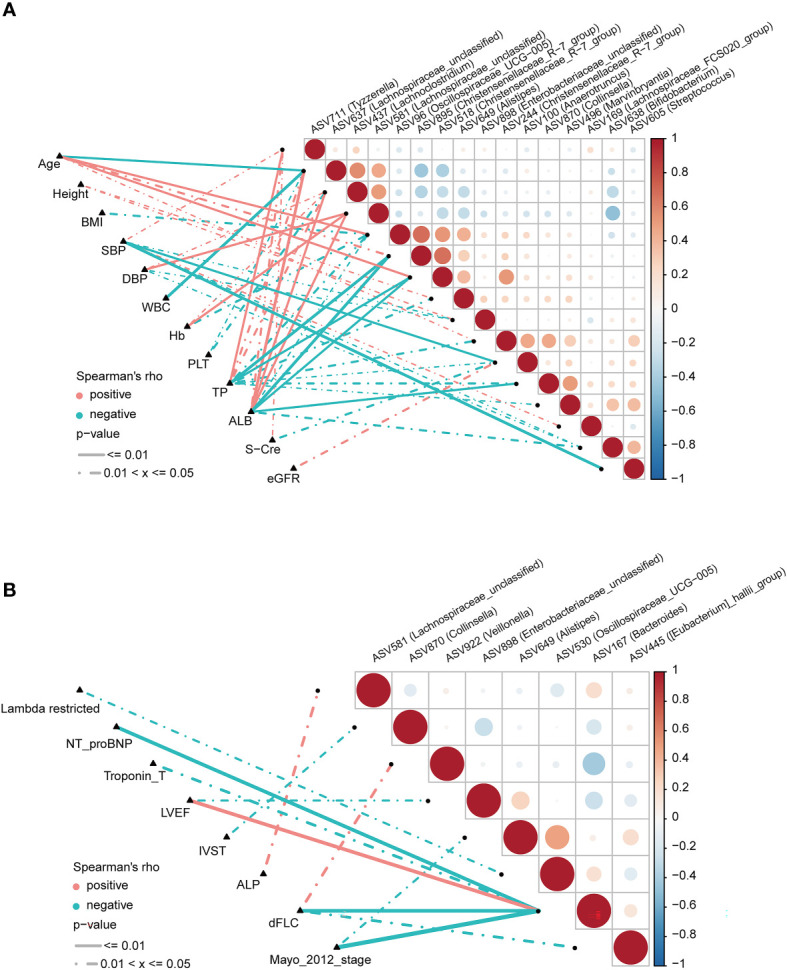
Correlation analysis of differential ASVs and clinical characteristics of AL amyloidosis patients by Spearman’s rank test. The oblique triangle heatmap indicated a taxon-taxon correlation between fecal microbiota and clinical parameters in **(A)** both AMY and HCs and **(B)** only AMY group (**Supplementary Tables 8, 9**). Positive values (red) indicate positive correlations. Negative values (blue) indicate inverse correlations. Solid lines represent *p* ≤ 0.01. Dotted lines represent 0.01 < *p* ≤ 0.05. AMY, patients with AL amyloidosis; HCs, healthy controls; ASVs, amplicon sequence variants. BMI, body mass index; dFLC, difference in the involved to uninvolved free light chain; DBP, diastolic blood pressure; SBP, systolic blood pressure; WBC, white blood cell; TP, total protein; ALB, albumin; Hb, hemoglobin; PLT, platelet; S-cre, serum creatinine; eGFR, estimated glomerular filtration rate; ALP, alkaline phosphatase; NT-proBNP, N-terminal pro-B-type natriuretic peptide; LVEF, left ventricular ejection fraction; IVST, interventricular septal thickness.

To explore correlations between ASVs and the clinical characteristics of patients with AL amyloidosis, Spearman’s rank correlation was performed. The oblique triangle heatmap indicated a taxon-taxon correlation between gut microbiota and clinical parameters. A total of 17 solid lines represented strong correlations (*p* < 0.01) between 9 ASVs and 7 clinical parameters and may be the focus of research **(** **Supplementary Table 8**; **Figure 6A** **)**. For example, ASV605 (*Streptococcus*) was negatively correlated with systolic blood pressure (SBP) and positively correlated with ASV244 (*Christensenellaceae_R-7_group*), ASV496 (*Marvinbryantia*) and ASV638 (*Bifidobacterium*). In the genus *Christensenellaceae_R-7_group*, ASV518 and ASV895 were negatively correlated with albumin (ALB) and total protein (TP), and ASV518 was also positively correlated with age.

As for AL amyloidosis-unique clinical parameters, a significant positive correlation existed between mayo 2012 stage and ASV649 (*Alistipes*) (ρ=-0.38, *p* = 0.049), and ASV167 (*Bacteroides*) (ρ=-0.61, *p* < 0.001). Moreover, lambda restricted had a negative correlation with ASV530 (*Oscillospiraceae_UCG-005*) (ρ=-0.39, *p* = 0.04). Significant positive correlations also existed between dFLC and ASV922 (*Veillonella*) (ρ=0.42, *p* = 0.027), and between ALP and ASV581 (*Lachnospiraceae_unclassified*) (ρ=0.40, *p* = 0.037, **Supplementary Table 9**; **Figure 6B**).

### 3.7 Identification and validation of ASV-based markers in the diagnosis of AL amyloidosis

**Figure 7:**
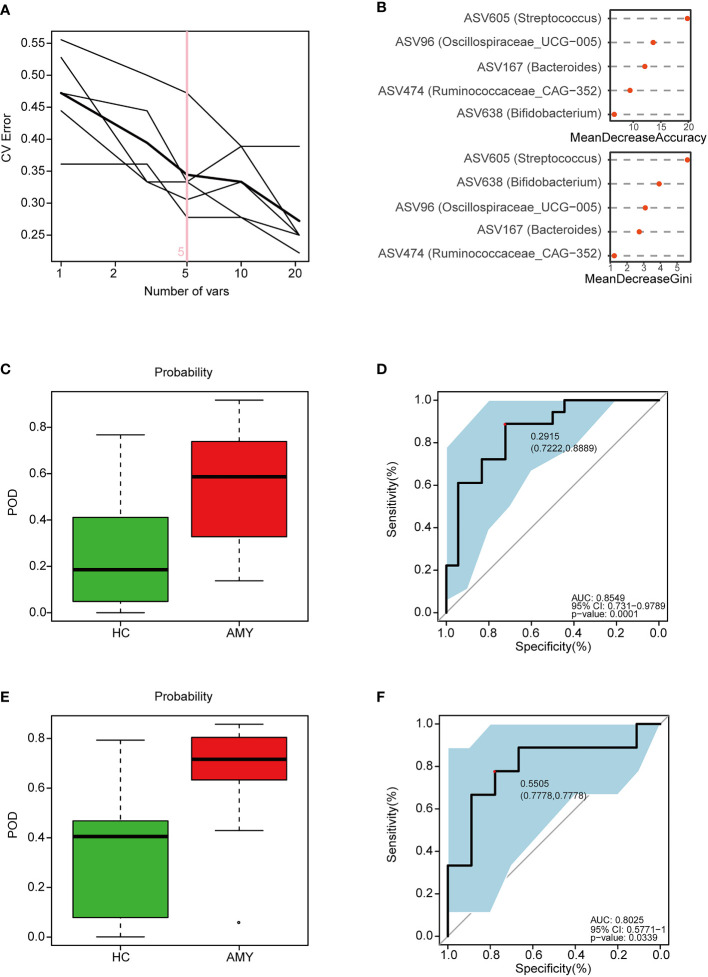
Identification and validation of microbial ASV-based markers of AL amyloidosis by random forest models. A total of 63 ASVs were used for random forest modeling and biomarker selection, which were significantly different between the two groups (Mann-Whitney U test, *p* < 0.05) and their corresponding abundance was more than 0.1% in any sample. **(A)** 5 ASVs were selected by random forest models as the optimal AL amyloidosis biomarkers (**Supplementary Table 10**). **(B)** Importance distribution map of the selected microbial markers in the model. **(C)** POD was significantly higher in AMY than HCs in the training set (**Supplementary Table 11**). **(D)** The POD index had an AUC = 0.8549 with a 95% CI = 0.731- 0.9789 between AMY and HCs in the training set. **(E)** POD was significantly higher in Eps than HCs in the test set (**Supplementary Table 12**). **(F)** The POD index had an AUC = 0.8025 with a 95% CI = 0.57771-1 between AMY and HCs in the test set. AMY, patients with AL amyloidosis; HCs, healthy controls; ASVs, amplicon sequence variants; POD, probability of disease; AUC, area under the curve.

According to the above significant difference in gut microbiota between AL amyloidosis and HCs, we assessed the potential use of gut microbiota-based signatures in the diagnosis of AL amyloidosis. A total of 63 ASVs were used for random forest modeling and biomarker selection, which were significantly different between the two groups (Mann-Whitney U test, *p* < 0.05) and their corresponding abundance was more than 0.1% in any sample. A five-fold cross-validation curve of the random forest model revealed that the 5 ASV-based markers were identified as the optimal marker set (**Figure 7A**). The relative abundance of the 5 ASV-based markers in each sample was presented in **Supplementary Table 10**. In the stochastic decision forest model, the distribution of ASV importance was demonstrated by the mean decrease in accuracy and mean decrease in the Gini coefficient (**Figure 7B**).

In the training phase, the probability of disease (POD) value was significantly increased in the AL amyloidosis samples compared with the control samples (*p* = 1.3×10^–4^, **Figure 7C**; **Supplementary Table 11**). The POD index achieved an AUC value of 0.8549 with 95% CI of 0.731 to 0.9789 between AL amyloidosis and HCs cohorts (*p* = 0.0001, **Figure 7D**).

In the validation phase, 9 HCs and 9 AL amyloidosis were used to validate the diagnostic efficacy of the POD for AL amyloidosis. Each POD of each patient was estimated and the relevant results are shown in the online **Supplementary Table 12**. The average POD value was significantly higher in the 9 patients with AL amyloidosis than that in 9 controls (*p* = 0.034, **Figure 7E**), and the POD attained an AUC value of 0.8025 (95% CI 0.5771-1, *p* = 0.0339) between AL amyloidosis and controls (**Figure 7F**).

### 3.8 Association between baseline gut microbiota and disease severity, treatment response, and prognosis in AL amyloidosis

Subgroup analysis was further done to reveal the association between the characteristics of gut microbiota at baseline and disease severity, treatment response, and prognosis in 27 patients with AL amyloidosis. All the following subgroups were matched between age, gender, and BMI (**Supplementary Table 13**).

#### 3.8.1 Association between baseline gut microbiota and heart involvement in AL amyloidosis

**Figure 8:**
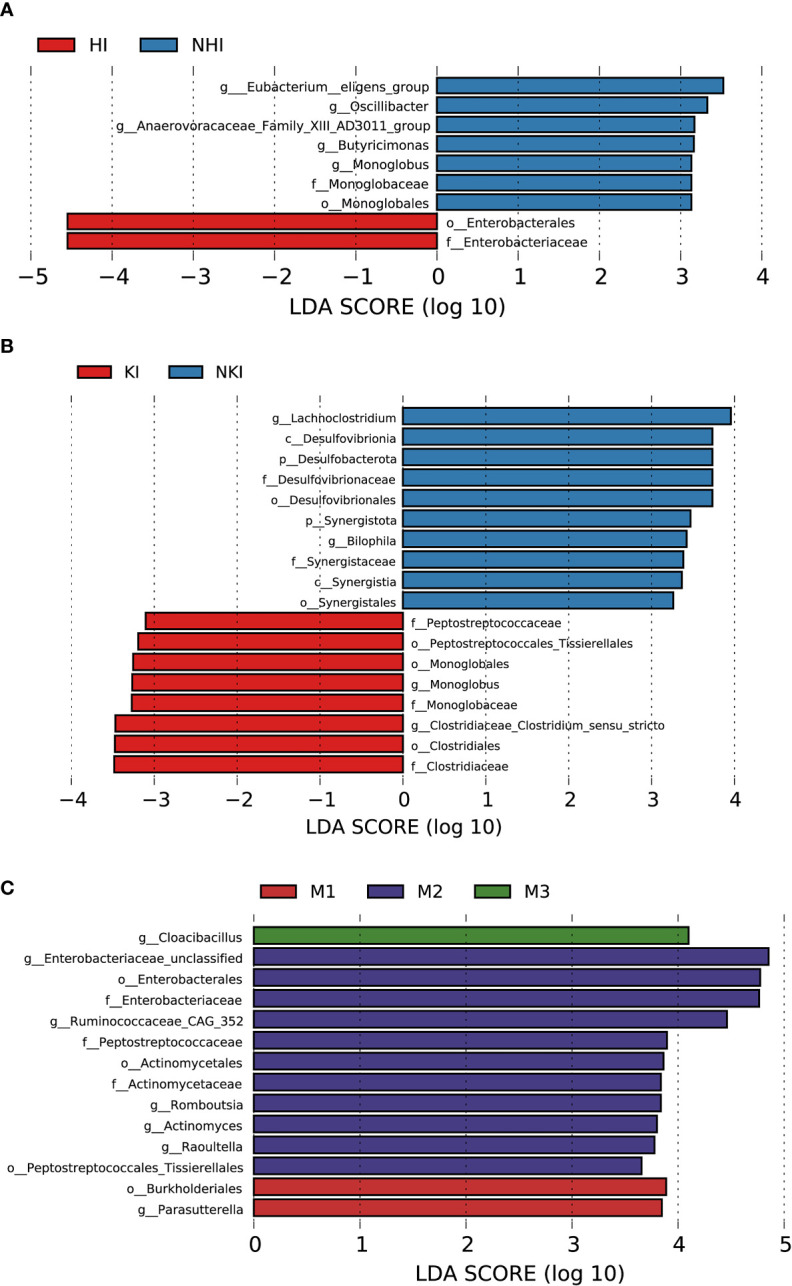
Association between baseline gut microbiota and disease severity in AL amyloidosis. Histogram of LDA scores calculated for selected taxa showed significant differences in microbe type and abundance between **(A)** HI and NHI, **(B)** KI and NKI, **(C)** M1, M2, M3 and M4 (**Supplementary Table 14**). LDA scores on a log10 scale are indicated at the bottom. The significance of the microbial marker increases with the LDA score. The default criteria was LDA > 2.5 and *p* < 0.05. All the subgroups were matched between age, gender, and BMI (**Supplementary Table 13**). AMY, patients with AL amyloidosis; LDA, linear discriminant analysis; HI, heart involvement; NHI, non-heart involvement; KI, kidney involvement; NKI, non-kidney involvement; M1, mayo 2012 stage I; M2, mayo 2012 stage II; M3, mayo 2012 stage III; M4, mayo 2012 stage IV.

Although patients with or without heart involvement did not have significant differences in the alpha and beta diversity in the baseline gut microbiota, they had abundance differences regarding some specific bacterial taxons. Compared with 10 patients without heart involvement, 17 patients with cardiac involvement had significantly enriched abundance of a bacterial taxon in the baseline gut microbiota, *o-Enterobacterales. f-Enterobacteriaceae* in phylum *Proteobacteria* (LDA > 2.5, *p* < 0.05, **Figure 8A**). While one bacterial taxon, *o-Monoglobales.f-Monoglobaceae.g-Monoglobus* was decreased in patients with cardiac involvement (in phylum *Firmicutes*, LDA > 2.5, *p* < 0.05, **Figure 8A**; **Supplementary Table 14**).

#### 3.8.2 Association between baseline gut microbiota and kidney involvement in AL amyloidosis

Compared with 5 patients without kidney involvement, 22 patients with kidney involvement had a higher abundance of three bacterial taxon clades in phylum *Firmicutes*, *o-Clostridiales.f-Clostridiaceae.g-Clostridiaceae_Clostridium_sensu_stricto, o-Monoglobales.f-Monoglobaceae.g-Monoglobus*, and *o-Peptostreptococcales Tissierellales.f-Peptostreptococcaceae*, and two main decreased abundance of bacterial taxon clades, *p-Desulfobacterota.c-Desulfovibrionia.o-Desulfovibrionales.f-Desulfovibrionaceae.g-Bilophila*, and *p-Synergistota.c-Synergistia.o-Synergistales.f-Synergistaceae* (LDA > 2.5, all *p* < 0.05, **Figure 8B**; **Supplementary Table 14**).

#### 3.8.3 Association between baseline gut microbiota and Mayo 2012 staging system in AL amyloidosis

In phylum *Proteobacteria*, order *Burkholderiales* and genus *Parasutterella* were enriched in patients with Mayo 2012 stage I. Two bacterial taxon chains were abundant in patients with Mayo 2012 stage II, *o-Actinomycetales.f-Actinomycetaceae.g-Actinomyces* and *o-Peptostreptococcales Tissierellales.f-Peptostreptococcaceae.g-Romboutsia.* Genus *Cloacibacillus* in phylum *Synergistota* was found enriched in patients with Mayo 2012 stage III (LDA > 2.5, all *p* < 0.05, **Figure 8C**). Patients with Mayo 2012 stage III/IV had a higher abundance of genus *Escherichia_Shigella* and *Ruminococcaceae_UBA1819*, while genus *Oscillospiraceae_UCG_003* and *Parasutterella* were decreased compared with patients with Mayo 2012 stage I/II (**Supplementary Figure 2**; **Supplementary Table 14**).

#### 3.8.4 Association between baseline gut microbiota and hematological response in AL amyloidosis

With a median follow-up of 19.0 months (range, 4.0 to 67.0), 19 (82.6%) of 23 patients had a hematologic response, including complete response (CR, 13 patients), very good partial response, (VGPR, 4 patients), partial response (PR, 2 patients) and 4 patients could not be evaluated because of incomplete data. According to the PCoA analysis based on weighted UniFrac distances, the microbial composition of CR patients was significantly distinct from VGPR patients (Adonis test, *p* = 0.0132, **Supplementary Figure 3A**). By applying LEfSe analysis (LDA > 2.5, **Supplementary Figure 3B**; **Supplementary Table 14**), we found one taxonomic chain, *o-Monoglobales.f-Monoglobaceae.g-Monoglobus* in phylum *Firmicutes* as well as genera *Eggerthella* and *Eubacterium hallii group* were specifically enriched in CR patients. In addition, two taxonomic chains, *p-Synergistota.c-Synergistia.o-Synergistales.f-Synergistaceae.g-Pyramidobacter* and *c-Firmicutes_Incertae_Sedis.o-Firmicutes_Incertae_Sedis_DTU014.f-Firmicutes Incertae Sedis DTU014.g-Firmicutes Incertae Sedis DTU014* were significantly enriched in VGPR patients, as well as genera *Eisenbergiella, Negativibacillus of* phylum *Firmicutes.* In phylum *Firmicutes*, we also found genera *Dorea* and *Epulopiscium* were abundant in PR patients.

**Figure 9:**
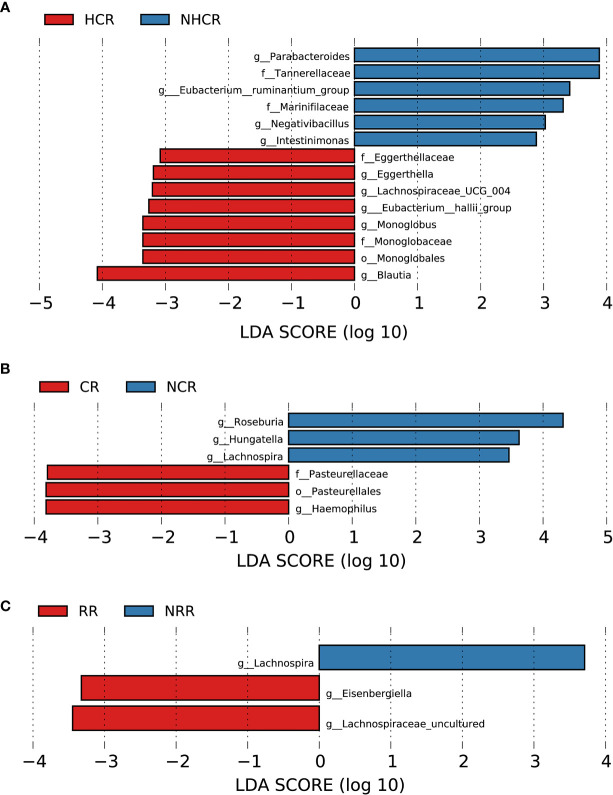
Association between baseline gut microbiota and hematological and organ response in AL amyloidosis. Histogram of LDA scores calculated for selected taxa showed significant differences in microbe type and abundance between **(A)** HCR and NHCR. **(B)** CR and NCR. **(C)** RR and NRR (**Supplementary Table 14**). LDA scores on a log10 scale are indicated at the bottom. The significance of the microbial marker increases with the LDA score. The default criteria was LDA > 2.5 and *p* < 0.05. All the subgroups were matched between age, gender, and BMI (**Supplementary Table 13**). AMY, patients with AL amyloidosis; LDA, linear discriminant analysis; HCR, hematological complete response; NHCR, no hematological complete response; CR, cardiac response; NCR, Non-cardiac response; RR, renal response; NRR, Non-renal response.

Patients were further divided into two groups based on whether they had CR or VGPR. Interestingly, consistent with the findings above in CR patients, a higher abundance of bacterial taxon clade, *o-Monoglobales.f-Monoglobaceae.g-Monoglobus*, and genera *Eggerthella* and *Eubacterium hallii group* were still enriched in 13 CR patients compared with 10 non-CR patients (**Figure 9A**). Genus *Eggerthella* was also enriched in 17 patients with VGPR or better compared with 6 patients less than VGPR (**Supplementary Figure 3C**; **Supplementary Table 14**).

By random forest, 5 microbial markers were identified for the prediction of hematological complete response (**Supplementary Figure 4A, B** **)**, including ASV97 (*Roseburia*), ASV183 (*Lachnospiraceae unclassified*), ASV352 (*Monoglobus*), ASV421 (*Lachnospira*), and ASV457 (*Clostridium innocuum group*). The AUC for the training set reached 0.9615(95% CI 0.8952-1, *p* = 0.0002, **Supplementary Figure 5A**).

#### 3.8.5 Association between baseline gut microbiota and cardiac and renal response in AL amyloidosis

In terms of organ response, a total of 13 and 18 patients could be evaluated for cardiac response and renal response, respectively. Patients in the cardiac response group (5 patients) had a higher abundance of one taxonomic chain, *o-Pasteurellales.f-Pasteurellaceae.g-Haemophilus* in phylum *Proteobacteria*, compared with patients with the cardiac response (8 patients). While three genera *Hungatella*, *Lachnospira*, and *Roseburia* of phylum *Firmicutes* were found enriched in the non-cardiac response group (**Figure 9B**, all *p* < 0.05, **Supplementary Table 14**). By random forest, 6 microbial markers were identified for the prediction of cardiac response (**Supplementary Figures 4C, D** **)**, including ASV147 (*Roseburia*), ASV412 (*Escherichia-Shigella*), ASV655 (*Bacteroides*), ASV752 (*Enterobacteriaceae unclassified*), ASV777 (*Odoribacter*), and ASV782 (*Roseburia*). The AUC for the training set reached 0.875(95% CI 0.6197-1, *p* = 0.0295, **Supplementary Figure 5B**).

Compared with patients without renal response, genera *Eisenbergiella* and *Lachnospiraceae uncultured* of phylum *Firmicutes* had a higher abundance in patients with renal response. While genus *Lachnospir*a of phylum *Firmicutes* was found abundant in patients without renal response (**Figure 9C**, all *p* < 0.05, **Supplementary Table 14**). By random forest, 2 microbial markers were identified for the prediction of renal response (**Supplementary Figures 4E, F** **)**, including ASV71 (*Lachnospira*) and ASV504 (*Faecalibacterium*). The AUC for the training set reached 0.9351(95% CI 0.8036-1, *p* = 0.0012, **Supplementary Figure 5C**).

#### 3.8.6 Association between baseline gut microbiota and the prognosis of patients with AL amyloidosis

**Figure 10:**
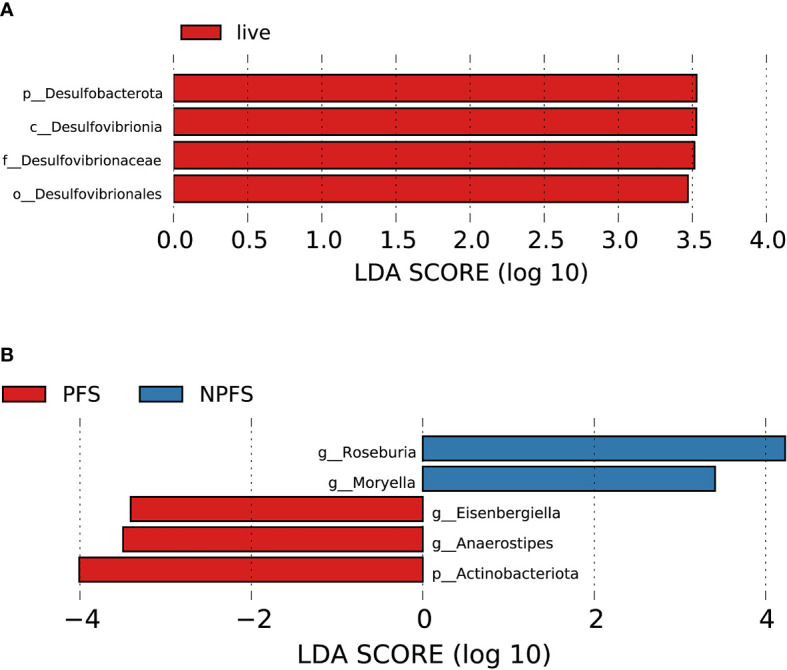
Association between baseline gut microbiota and prognosis in AL amyloidosis. Histogram of LDA scores calculated for selected taxa showed significant differences in microbe type and abundance between **(A)** die and live group; **(B)** PFS and NPFS group (**Supplementary Table 14**). LDA scores on a log10 scale are indicated at the bottom. The significance of the microbial marker increases with the LDA score. The default criteria was LDA > 2.5 and *p* < 0.05. All the subgroups were matched between age, gender, and BMI (**Supplementary Table 13**). AMY, patients with AL amyloidosis; LDA, linear discriminant analysis; PFS, progression-free survival; NPFS, patients without progression-free survival.

During the follow-up time, four patients died, who had a lower abundance of one bacterial taxon chain, *p-Desulfobacterota.c-Desulfovibrionia.o-Desulfovibrionales.f-Desulfovibrionaceae* (**Figure 10A**). Patients without progression-free survival had a higher abundance of genus *Roseburia* and *Moryella* in phylum *Firmicutes*, but a decreased abundance of genus *Anaerostipes* and *Eisenbergiella* of phylum *Firmicutes* and phylum *Actinobacteriota* (**Figure 10B**, **Supplementary Table 14**). By random forest, 2 microbial markers were identified for the prediction of progression-free survival (**Supplementary Figures 4G, H** **)**, including ASV584 (*Anaerostipes*) and ASV823 (*Eisenbergiella*). The AUC for the training set reached 0.9412(95% CI 0.8259-1, *p* < 0.0001, **Supplementary Figure 5D**).

## 4 Discussion

In this study, we used 16S rRNA sequencing to analyze the gut microbiota of newly diagnosed patients with AL amyloidosis. Although no significant differences in community richness and diversity were observed between AL amyloidosis and HCs, significant differences were found in bacterial composition between the two groups. Notably, this study was also the first to successfully establish and validate a diagnosis model based on microbial ASV markers for AL amyloidosis. Further subgroup analysis revealed the association between baseline gut microbiota and disease severity, treatment response, and prognosis of patients with AL amyloidosis.

Emerging evidence revealed that different diseases have relatively distinct microbial profiles and gut microbial alteration is unique for each disease, such as MM (8), Alzheimer’s disease (34), and FMF-related AA amyloidosis (13). In this study, we found treatment naïve AL amyloidosis patients may possess a specific profile of gut microbiota characterized by composition changes at different levels and several enriched taxonomic chains. Notably, phylum *Actinobacteriota* as a whole, as well as several families and genera classified within *Actinobacteriota* were found increased in patients with AL amyloidosis. Specifically, the enrichment of genera *Bifidobacterium*, *Collinsella* and *Eggerthella* resulted in a subsequent higher abundance of phylum *Actinobacteriota*. Recently, mounting evidence showed *Bifidobacterium* can modulate immune system by increasing immunoglobulins, and inducing or reducing pro- or anti-inflammatory cytokines, respectively (35–37). And it is widely assumed that immunoglobulin light chains are precursors for amyloid deposits in AL amyloidosis. Accordingly, we speculated that a higher abundance of genus *Bifidobacterium* might have a promoting effect on the induction of immunoglobulin production, further facilitating the development of AL amyloidosis.

The increase of phylum *Verrucomicrobia* is most likely attributed to the increase of genus *Akkermansia* in AL amyloidosis. In general, *Akkermansia muciniphila* has been found to be significantly linked to a variety of physiological processes, including glucose and lipid metabolism, as well as immune response (38). A study claimed that the relative abundance of *Akkermansia muciniphila* in mice with colitis was positively correlated with injured histology and colonic inflammation (39) while Kang et al. found extracellular vesicles from *Akkermansia muciniphila* were significantly protective in DSS-treated mice (40). The increase of genus *Akkermansia* in AL amyloidosis was probably one of the mechanisms of self-protection against gut dysfunctions. The relationship between genus *Akkermansia* and the pathogenesis of AL amyloidosis is still unknown and worth further investigation and confirmation.

In addition to the above two increased phyla, we also found a decrease of phylum *Bacteroidota* in AL amyloidosis. *Bacteroidota* is the largest phylum of Gram-negative bacteria inhabiting our gastrointestinal tract and is regarded as the key player in the healthy state and sophisticated homeostasis of gut microbiota (41). Besides, to our knowledge, specific roles have been attributed to some genera of phylum *Bacteroidota* in the development of human diseases such as obesity, diabetes mellitus, rheumatoid arthritis, atherosclerosis, and neurodegenerative diseases (42–46). Further studies are needed to understand the potential relationship between the decrease of phylum *Bacteroidota* and the development of AL amyloidosis.

Interestingly, consistent with the previous study in MM (8), the nitrogen-cycle-bacteria such as the genus *Streptococcus* of phylum *Firmicutes*, was also markedly higher in AL amyloidosis. During the development of MM, increased urea or NH4+ and descending renal function led to the preferred growth of nitrogen-cycle-bacteria. Subsequently, urea is hydrolyzed effectively and utilized to synthesize L-glutamine, which is transferred to the host, hence hastening the progression of MM (8). In our study, ASV605 (*Streptococcus*) was found to be negatively correlated with SBP and positively correlated with the other three ASVs (ASV244, ASV496, and ASV638). We further identified ASV605 (*Streptococcus*) as the most important ASVs of five microbial markers for the diagnosis of AL amyloidosis. Thus, the genus *Streptococcus* may also contribute to the development of AL amyloidosis. Additionally, it is widely known that clonal plasma cells are both presented in AL amyloidosis and MM, and connected these two diseases. Therefore, the impact of gut microbiota on the proliferation of clonal plasma cells may be the focus of future researches. More importantly, the successful validation of their diagnostic efficacy in our cohort further made us believe that gut microbiota-based biomarkers might be valuable non-invasive methods for the diagnosis of AL amyloidosis. Further metagenomic sequencing of targeted species may strengthen the diagnostic efficiency of AL amyloidosis.

Furthermore, we observed significantly different functional abundance of KEGG pathways in patients with AL amyloidosis compared to HCs, which revealed that alterations in the abundance of certain gut microbial species may play a crucial role in regulating metabolic functions and inflammatory response. However, as the first research on gut microbiota in AL amyloidosis, other relevant researches are vacant and their related pathogenesis in this disease is still unclear. Additionally, spearman’s analysis revealed the potential correlations between gut microbiota and clinical features. All the above findings need further validation and have laid the foundation for more research on the role of gut microbiota in the development of AL amyloidosis.

With the median follow-up of 19.0 months, some gut microbiota at baseline had been found to be associated with disease severity, treatment response, and prognosis of AL amyloidosis patients. Although ASV-based microbial markers were also identified as potential tools to predict the outcome of AL amyloidosis, further investigation and validation are needed due to the small number of subgroups.

Despite the valuable findings, our study still had certain shortcomings. First, due to the rarity of AL amyloidosis and the strict inclusion criteria, we recruited a relatively small sample size, which resulted in limited number of patients in further subgroup analysis. In order to fully investigate the impact of gut microbiota and further confirm our results, a larger cohort of patients with varying stages of illness is needed. Second, some confounding factors such as dietary habits, should also be taken into account. Due to the average age being too large in these two groups, it is hard to find absolutely healthy people and the mean BMI of the healthy group was slightly high. Moreover, participants in our study were all from China, and a more comprehensive study should be conducted and validated on a larger random sample of individuals from different regions. Third, we only evaluated fecal microbiota, which didn’t adequately represent the whole profiles of mucosal microbiota.

In conclusion, for the first time, we highlighted the difference in gut microbiota between AL amyloidosis and HCs based on ASVs. The increase of *Actinobacteriota* and *Verrucomicrobiota* and decrease of *Bacteroidota* may contribute to the development of AL amyloidosis. Moreover, our study is the first to successfully establish and validate the ASV-based microbial diagnostic model of AL amyloidosis in China. Interestingly, some gut microbiotas at baseline are associated with disease severity, treatment response, and prognosis of AL amyloidosis patients, which needed further investigation and validation. As the first report of the gut microbiota in AL amyloidosis, it injected its own strength into the ocean and opened new avenues for more studies about microbe-based strategies of diagnosis and treatment in patients with AL amyloidosis in the future.

## Data availability statement

The original contributions presented in the study are included in the article/**Supplementary Material**, further inquiries can be directed to the corresponding author/s.

## Ethics statement

The studies involving human participants were reviewed and approved by Ethics Committee of Xijing Hospital of the Fourth military medical university (KY20192070). The patients/participants provided their written informed consent to participate in this study. Written informed consent was obtained from the individual(s) for the publication of any potentially identifiable images or data included in this article.

## Author contributions

JY, SS, and JZ conceived and designed this study. JZ, XN, ZY, W-FG, YQ, YX, QJ, YW, RM, CL, MZ and BH participated in data acquisition, analysis, and interpretation. JY and JZ drafted the manuscript. All authors contributed to the article and approved the submitted version.

## Funding

This study was sponsored by grants from Xijing hospital discipline promoting plan (Reference number: XJZT21L15), National Natural Science Foundation of China grants (Reference number: 82170722, 81870470), Key project of Shaanxi province (Reference number: 2017ZDXM-SF-045).

## Acknowledgments

We thank all the generous volunteer subjects who were enrolled in the study.

## Conflict of interest

The authors declare that the research was conducted in the absence of any commercial or financial relationships that could be construed as a potential conflict of interest.

## Publisher’s note

All claims expressed in this article are solely those of the authors and do not necessarily represent those of their affiliated organizations, or those of the publisher, the editors and the reviewers. Any product that may be evaluated in this article, or claim that may be made by its manufacturer, is not guaranteed or endorsed by the publisher.
