# Supplementary material for: Alterations of the gut microbiota in patients with immunoglobulin light chain amyloidosis
Source: Front Immunol. 2022 Oct 19;13:973760. doi: 10.3389/fimmu.2022.973760 (PMC9628213; doi:10.3389/fimmu.2022.973760)
Supplement: Supplementary file 1 [file DataSheet_1.pdf]

frontiers

Supplementary Material

1.1 Supplementary Figures

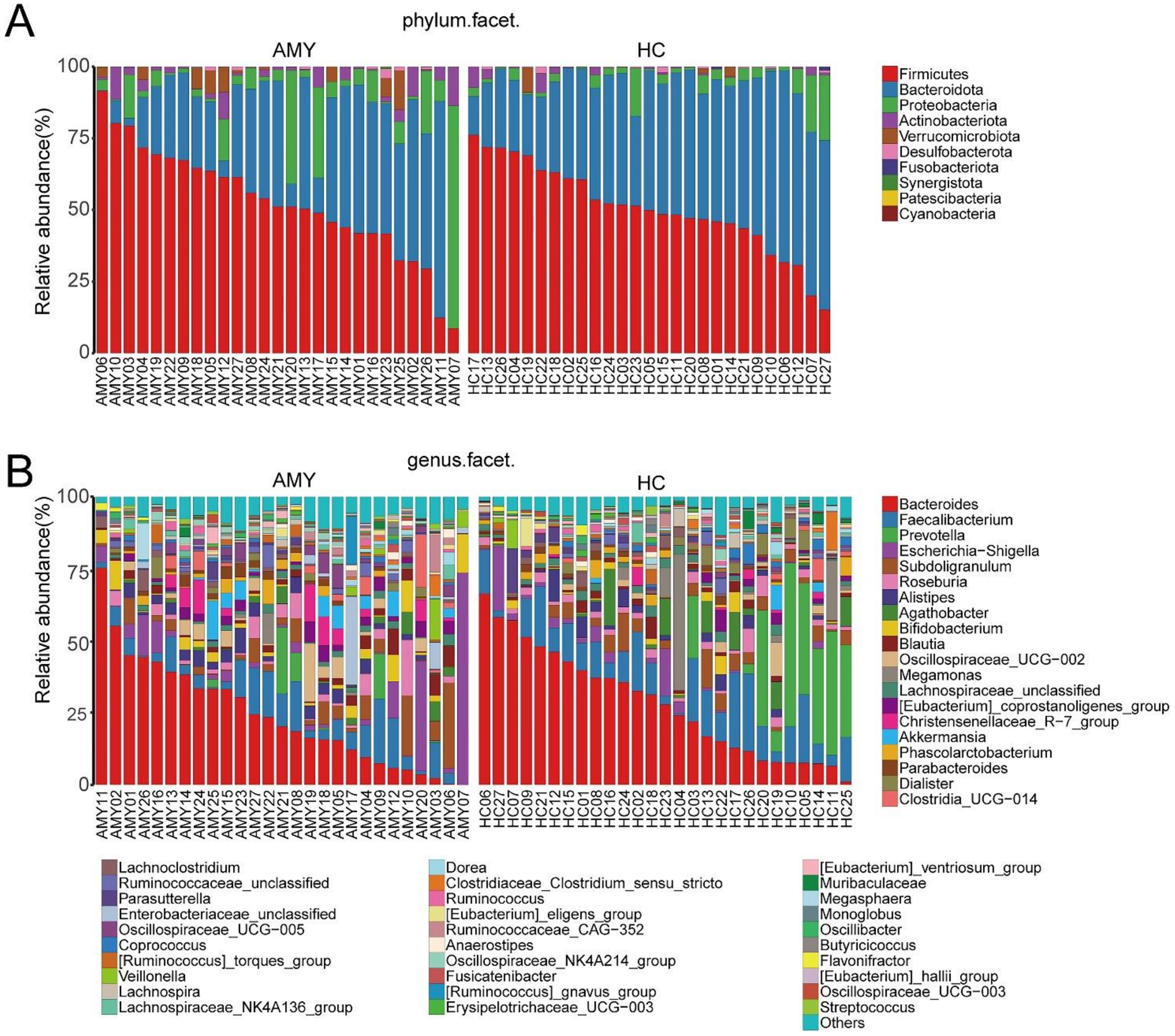

Supplementary Figure 1. The **(A)** phylum and **(B)** genus levels of fecal microbial composition in each sample from two groups. **AMY**, patients with AL amyloidosis; **HC**, healthy control.

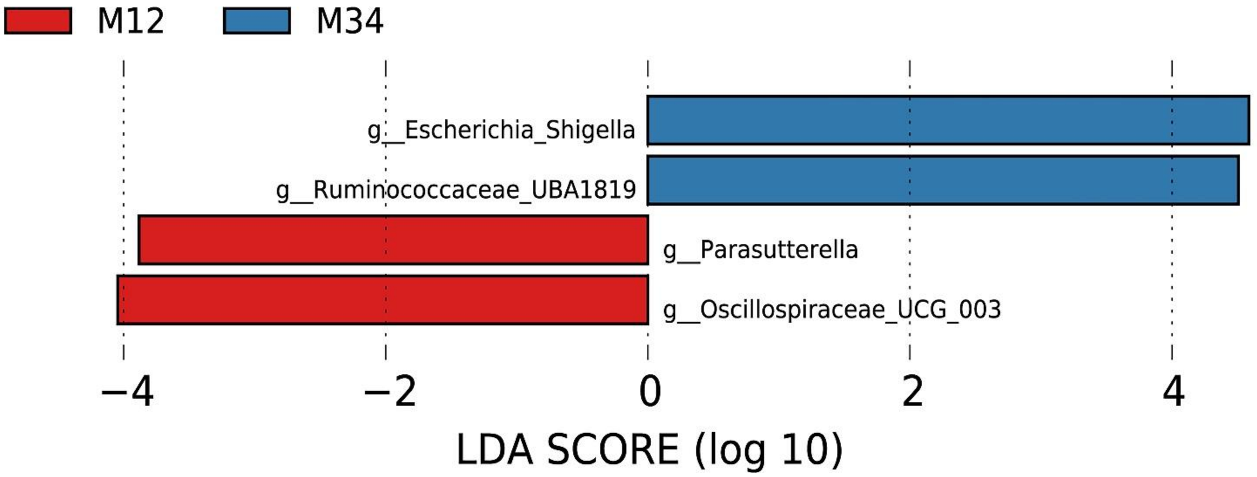

Supplementary Figure 2. Histogram of LDA scores calculated for selected taxa showed significant differences in microbe type and abundance between M12 and M34 groups. The significance of the microbial marker increases with the LDA score. The default criteria were LDA > 2.5 and *p* < 0.05. **LDA**, linear discriminant analysis; **M12**, mayo 2012 stage I/II; **M34**, mayo 2012 stage III/IV. **g**, genus

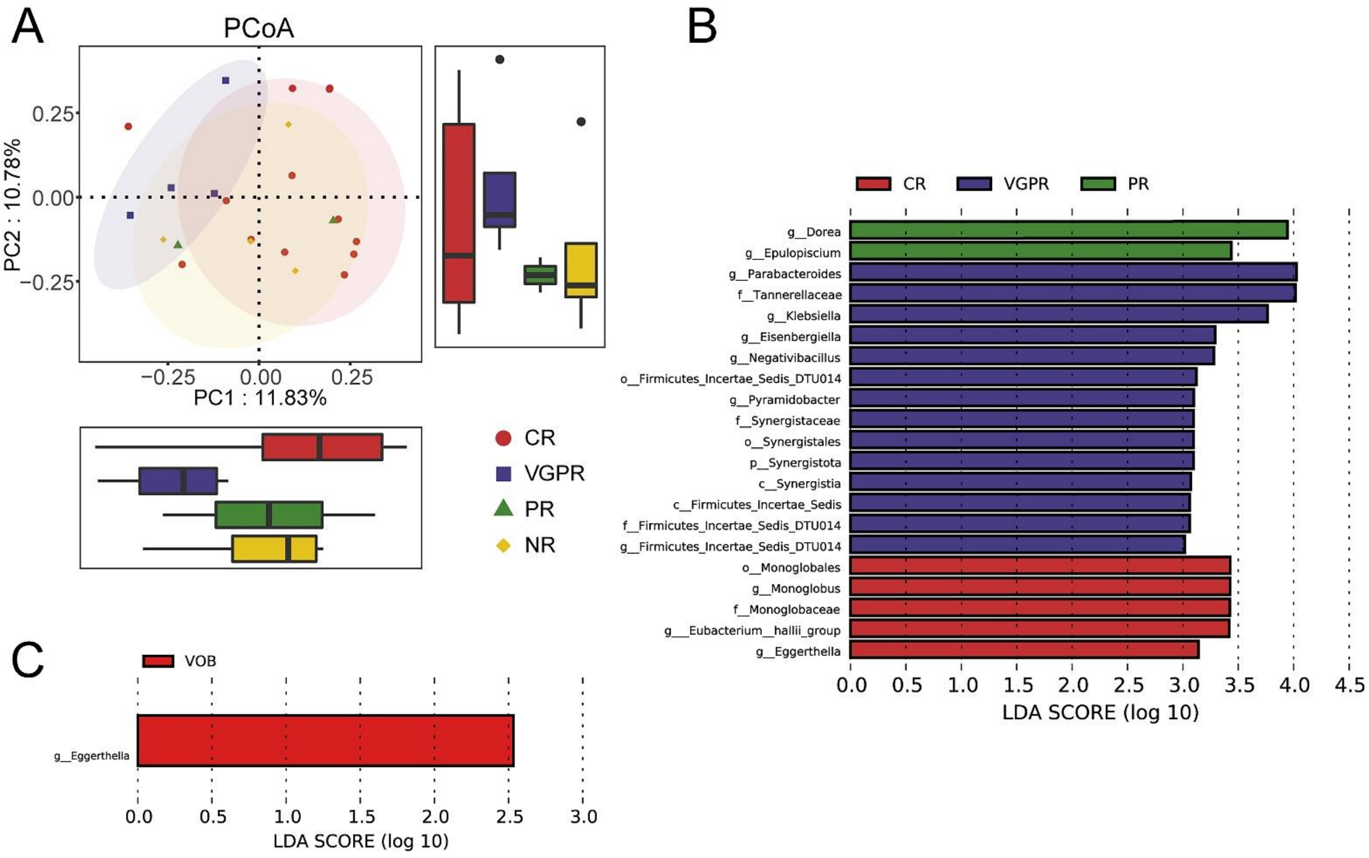

Supplementary Figure 3. Baseline gut microbiota related to hematological response in AL amyloidosis. **(A)** PCoA analysis based on weighted UniFrac distances, the microbial composition of CR patients was significantly distinct from VGPR patients (Adonis test, *p* = 0.0132). Histogram of LDA scores calculated for selected taxa showed significant differences in microbe type and

abundance between **(B)** CR, VGPR, PR, NR; **(C)** ≥VGPR or not. The default criteria were LDA > 2.5 and $p < 0.05$. All the subgroups were matched between age, gender, and BMI (**Supplementary Table 13**), and significant different taxa with calculated LDA scores between patients in different subgroups were shown in **Supplementary Table 14**. **PCoA**, principal coordinate analysis; **LDA**, linear discriminant analysis; **CR**, complete response; **VGPR**, very good partial response; **PR**, partial response; **NR**, no response; **LTV**, less than VGPR.

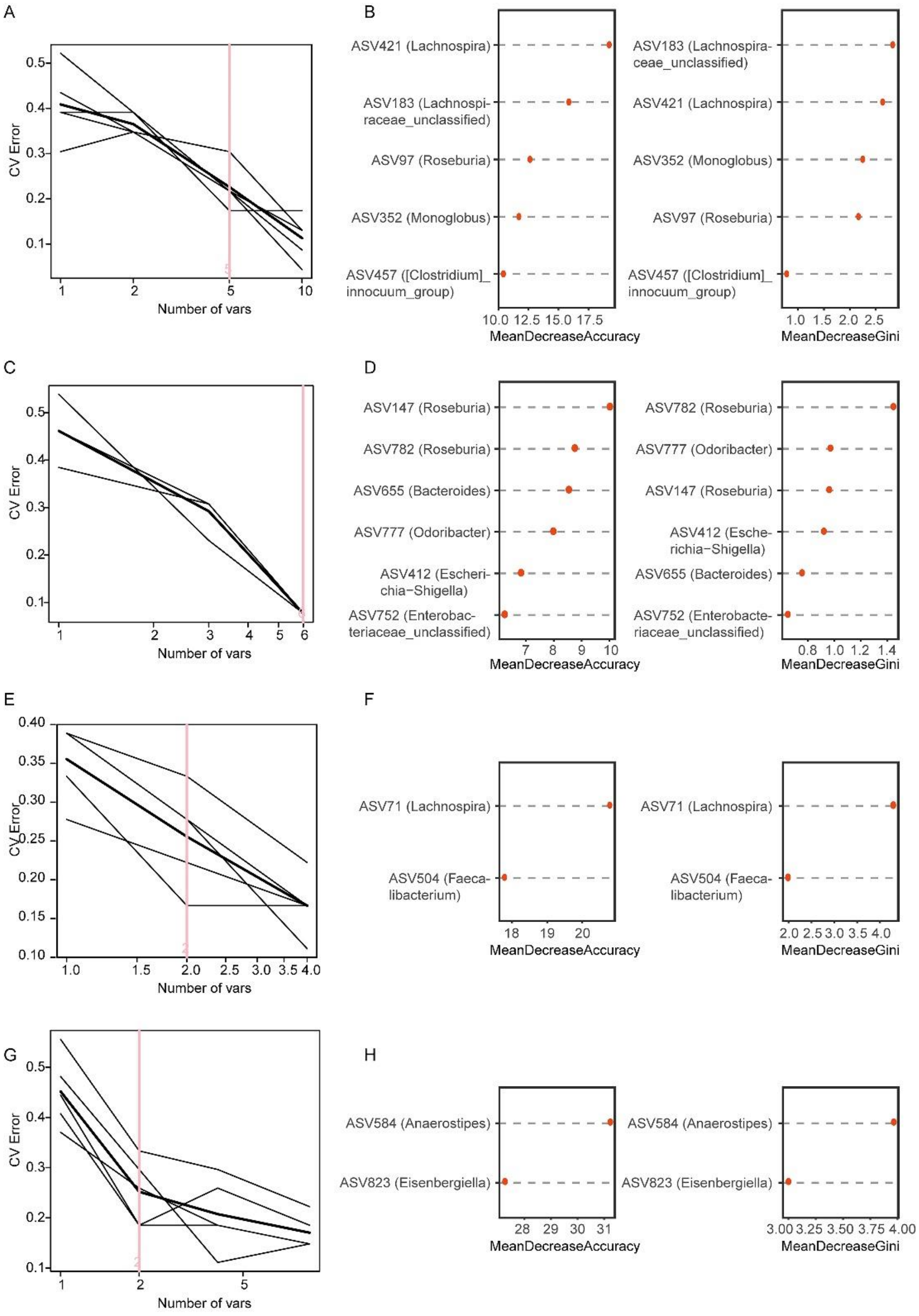

Supplementary Figure 4. Selection of ASV-based microbial markers in predicting the outcome of AL amyloidosis by random forest. A cross-validation curve of the random forest model revealed that 5, 6, 2, and 2 ASV-based microbial markers for the prediction of **(A)** hematological complete

response, **(C)** cardiac response, **(E)** renal response, and **(G)** progression-free survival, respectively. The distribution of the corresponding ASV importance was demonstrated by the mean decrease in accuracy and mean decrease in the Gini coefficient in the model of **(B)** hematological complete response, **(D)** cardiac response, **(F)** renal response, and **(H)** progression-free survival, respectively. **ASV**, Amplicon sequence variant.

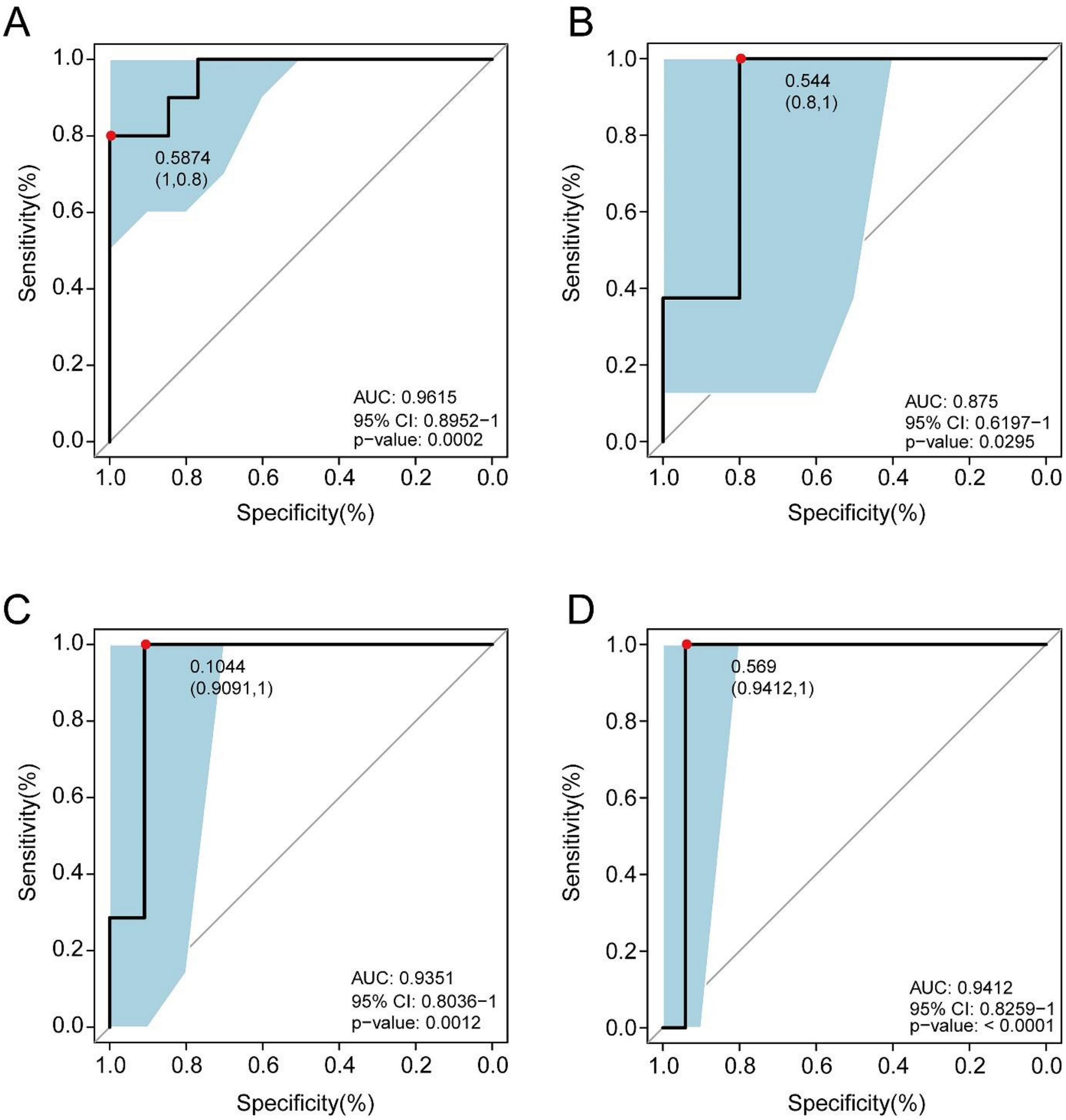

Supplementary Figure 5. Identification of ASV-based microbial markers in predicting the outcome of patients with AL amyloidosis. A total of 5, 6, 2, and 2 microbial markers were identified for the prediction of hematological complete response, cardiac response, renal response, and progression-free survival, respectively (**Supplementary Figure 4**).The AUC for the training set reached 0.9615(95% CI 0.8952-1, $p = 0.0002$), 0.875(95% CI 0.6197-1, $p = 0.0295$), 0.9351(95% CI 0.8036-1, $p = 0.0012$), and 0.9412(95% CI 0.8259-1, $p < 0.0001$) for **(A)** hematological complete response, **(B)** cardiac

response, **(C)** renal response and **(D)** progression-free survival, respectively. **ASV**, amplicon sequence variant; **AUC**, area under curve.
